# Factors associated with school achievement of children aged 8–10 years in rural Bangladesh: Findings from a post hoc analysis of a community-based study

**DOI:** 10.1371/journal.pone.0254693

**Published:** 2021-07-28

**Authors:** Sheikh Jamal Hossain, Fahmida Tofail, Hasan Mahmud Sujan, Shams El Arifeen, Jena Hamadani

**Affiliations:** 1 Maternal and Child Health Division (MCHD), icddr,b, Dhaka, Bangladesh; 2 Department of Women’s and Children’s Health, Faculty of Medicine and Pharmacy, Uppsala University, Uppsala, Sweden; University of Padova, ITALY

## Abstract

**Background:**

Education is one of the most important human capitals. Investment in education at early age returns best. A lot of factors influence children’s educational achievement. Studies in developed countries well established the relation of school achievement with its associated variables. But information is lack on what factors play important role for school achievement at early age in low resource settings like Bangladesh. We aimed to find factors associated with school achievement in rural Bangladesh.

**Method:**

The data were acquired from a long-term follow up study, conducted in 8–10 years old children (n = 372). We used a locally developed school achievement tool based on Wide Range Achievement Test-4 to measure reading, spelling and math computation, Wechsler abbreviated scale of intelligence to measure intelligence Quotient (IQ), Digit span forward and backward for short term memory, and locally available Strength and Difficulties Questionnaire to measure behaviour. Socioeconomic and anthropometric information of the mothers and children were also collected. Multicollinearity of the data was checked. Unadjusted and adjusted multiple linear regression analysis was performed.

**Findings:**

Years of schooling and short-term memory were positively related to reading, spelling and math computation. For years of schooling it was-reading B = 8.09 (CI 5.84, 10.31), spelling 4.43 (4.33, 8.53) and math computation 5.23 (3.60, 6.87) and for short term memory- reading 3.56 (2.01,5.05), spelling 4.01 (2.56, 5.46) and math computation 2.49 (1.37, 3.62). Older children had lower scores of reading -0.48 (-0.94, -0.02), spelling -0.41 (-0.88, -0.02) and math computation -0.47 (-0.80, -0.14). Children’s IQ predicted reading 0.48 (0.14, 0.81) and spelling 0.50 (0.18, 0.82) skills. Mother and father’s education predicted Spelling 0.82 (0.16, 1.48) and reading 0.68 (0.06, 1.30) capacity respectively. Children enrolled in private schools had higher reading 10.28 (5.05, 15.51) and spelling 6.22 (1.31, 11.13) than those in the government schools. Children with more difficult behaviour tended to have lower scores in reading -0.51 (-0.96, -0.05).

**Conclusion:**

Children’s school achievement is influenced by their IQ, years of schooling, type of school and parents’ education. Therefore, intervention should be made to focus specifically on these variables and establish the effect of this intervention through robust research design.

## Introduction

Education sector is very important for policy makers especially for low- and middle-income countries since investment in health and education is positively associated with improved human capital and rapid economic growth [1]. Education is one of the key contributors for economic development [2]. Education reflects occupational and social success in adult life [3]. Many studies documented that school achievement is related to socioeconomic status e.g. mother’s education, wealth status [4–6]. Socioeconomic status e.g maternal education, quality of housing was found independent predictor for school achievement [7]. A recent meta-analysis was performed based on 215,649 students from 78 independent samples in the basic education stage conducted in China on the relation between socioeconomic status and academic achievement and it found a moderate relation [8]. Other studies reported that children’s academic achievement is influenced by children’s nutritional [9, 10] and developmental status [11–13]. A meta-analysis using 240 independent samples proved that children’s school achievement is associated with their intelligence [14]. Studies also reported that children’s school achievement is associated with school factors e.g. years of schooling, type of school, teacher-students relationship, curricula of the school. [15–17]. Nevertheless. government policy and commitment play an important role to access to primary education especially for the poor and to improve the quality of education [18–19].

Like many other developing countries, children’s growth, development and education are major concerns in Bangladesh [20]. Bangladesh has well achieved education related Millennium Development Goal (MDG) by 2015. In addition to that achievement, the country has now focused on quality education especially in pre-primary and primary education [21].

Achievement of optimum quality in education is a big challenge for any settings since school achievement depends on many aspects e.g. family’s socioeconomic and school factors, children’s health and nutrition and developmental status.

In developing countries including Bangladesh, there is scarce information on which factors are independently associated with children’s academic achievement when considered multi aspects of school achievement. We considered children’s background characteristics, nutrition, development and behavioural status, school factors, home stimulation offered for the children and maternal nutritional status in this study to identify what factors are associated with school achievement in rural Bangladesh.

## Methods

### Participants

We collected this information from a follow-up of a cluster randomized controlled trial (cRCT) conducted in 2015. The original cRCT was conducted in 2006. At enrolment of the original study, the children were recruited at 6–24 months from 32 villages of Monohordi sub district in rural Bangladesh. Half of the children were stimulated and another half did not receive any stimulation. The stimulated children received psychosocial stimulation e.g. puzzles, picture books and play materials. The mothers of the children were trained to stimulate the children. In both the stimulated and no stimulated group of children, there were children with iron deficiency anemia and non-anemic children. All anaemic children received iron therapy for six months. After three months of completion of iron therapy, anaemia status was checked and anemia in all children but one was corrected. Non-anemic children who received psychosocial stimulation were significantly benefitted [B ± SE = 5.7 ± 1.9 (95% CI: 2.0, 9.4), *P* = 0.003] in mental development compared to non-stimulated children. There was no effect on anemic children’s development or all on children’s motor development and behaviour [22].

Almost 8 years later, we traced 372 out of 434 children of this cohort when children’s age was around 8 years in 2015 and this paper reports on that data. The participants were recruited from thirty villages of eight Unions of Monohordi sub-district. Children having special education needs were excluded from the study as these children’s performance may under report the school achievement score. The education system of this study area is similar to other rural areas of Bangladesh where there are government schools, private schools and Madrasa but no English medium schools. Madrasa is a special type of education system in Indian sub-continent which is generally based on Islamic religion and its components: Quran and Hadith with a combination of general education system. There were no English medium schools in the study area. In Bangladesh, compulsory primary education is provided by the government free of cost. Recruitment process of the participants at enrollment in 2006 is described in details elsewhere [22].

At this stage the mean (SD) age of the children was 8.37 (0.65) years. The Mean (SD) years of schooling of the children was 3.71 (1.12) years. Most 251 (68.60%) of the children were studying in government primary schools while 73 (16.8%) and 42 (9.7%) were in private schools and madrasa respectively. The mean (SD) mother and father’s years of education of the children were 5.32 (3.37) and 4.66 (3.81) respectively.

### Measurements

A locally developed school achievement test based on Wide Range Achievement Test 4th version (WRAT4) [23] was used to measure reading, spelling and math computation. The tools have previously been used in Bangladesh [24, 25]. The Wechsler abbreviated scale of intelligence (WASI-II) was selected as a measure of children’s IQ. WASI has been used by the Child Development Unit, icddr,b in Bangladesh previously and was culturally adapted before use [25]. Digit span [26] forward and backward were used to measure children’s short-term working memory and executive function. It has also been used in Bangladesh previously [24]. The behaviour of the children was assessed using of the locally available tools developed based on Strength and Difficulties Questionnaire (SDQ) for children aged 6 to 10 years. The original tool is used internationally to assess behavioural strengths and problems with its prosocial and difficulties scales [27]. This tool has been used in previous studies in Bangladesh [28]. Stimulation and support the child received from the home environment was assessed with the Middle Childhood Home Observation for the Measurement of the Environment (MC-HOME) [29]. Height, weight and Mid Upper Arm Circumference (MUAC) of the children and mothers were measured using standard procedure [30]. Children’s OFC were collected from the original cRCT. Socio demographic and economic information were collected from the follow-up of the cRCT. We described above measurements details in the main manuscript. We conducted another survey on this population after about 6 months and collected the information on years of schooling and type of schools.

The questionnaires used for data collection were adapted and used in the studies of icddr,b Child Development Unit. The adapted version of WASI II was piloted on 52 children, and intraclass correlations for test-retest reliabilities ranged between 0.77 and 0.88. The locally available scholastic achievement test was administered to 52 children, and test-retest reliabilities ranged between 0.82 and 0.98. The Number Stroop test for executive function showed test-retest reliability scores of 0.74 and 0.79.

The locally available tools developed based on Strength and Difficulties Questionnaire (SDQ) was validated in which the test-retest reliability of total SDQ was r = 0.82 and ranged from 0.67 to 0.86 for its subscales (A Hilaly, S Shiraji, F Mehrin, J Hamadani, F Tofail, S Huda, unpublished results, 2008).

The assessors were trained by a trainer, who has a master degree in psychology with 10 years experiences in child development measurement tools. Before starting the measurements on study children, each assessor practiced on at least 5 non-study children and then assessed 10 non-study children in front of the trainer who also rated the child independently. The data was calculated for interobserver reliabilities of the assessor with the trainer and when interobserver reliability reached a minimum of 0.80 then the assessors were considered eligible to collect data from the field.

### Data entry, processing and analysis

Data were checked for completeness before data entry and the inconsistent data were re-checked with the hard copy of the questionnaire. The data was then entered into SPSS (version 21) cleaned and coded. The data cleaning process included running a simple frequency after data entry for its consistency to fix or remove incorrectly formatted, duplicate, or incomplete data within our dataset. Data were recoded to analyze as per requirement. Crowding index was constructed by dividing number of people by number of bed rooms. Housing index was calculated considering construction materials used for roof, walls, and floor of the house. Higher prices of construction materials were scored higher. So, higher score of housing index meant better housing. Anthropometric information of the children was converted to height-for-age Z score (HAZ), weight-for-age Z score (WAZ) and body mass index-for-age Z score (BAZ) of WHO AnthroPlus (version 1.0.4) and compared with reference data according to WHO 2006 population. Mothers’ Body Mass Index (BMI) was calculated using the formula ‘weight in kilograms divided by height in meters squared’.

Before analysis, the normality of main outcomes was checked. Descriptive statistics was used to present proportion, mean and standard deviation in tabulation form. Multicollinearity of the covariates was tested. We dropped BAZ from the analysis since it showed multicollinearty with WAZ of the children. Pearson correlation was done to see the relationship between the variables. Unadjusted linear regression analysis was done to assess the relationship between covariates and outcome variables separately. Then we conducted three multiple linear regression analysis considering reading, spelling and math computation as dependent variables in each analysis. The adjustment included three types of independent variable groups separately: i) background characteristics including school factors ii) Anthropometry of the children and the mothers and iii) Developmental status of the children.

Finally, all the three groups of variables were adjusted in a single model to explore significant predictors for reading, spelling and math computation following a backward elimination method. In the final model, we also considered variables having biological and scientific plausibility with main outcomes. Anemia and psychosocial stimulation, the intervention of the original study during infancy, was controlled in the finally adjusted multiple linear regression analysis. In all cases p-value less than 0.05 were accepted as statistically significant.

### Ethical considerations

The study protocol, consent forms, and data collection instruments were reviewed and approved by the Institutional Review Board of icddr,b (Protocol Number: PR-15059) and informed written consent was obtained from the parents or primary caregivers of the children.

## Results

In total 372 children participated. The Mean (SD) reading, spelling and math computation scores were 92.06 (23.21), 85.25 (21.89) and 77.29 (15.93). Table 1. Girls, children of higher educated parents and children from private schools had higher reading, spelling and math computation score (S1 Fig).

**Table 1:** Children’s developmental status and school performance scores (n = 372).

Characteristics#	Mean / Numbers N = 372	SD / %
**Developmental scores of the children**		
FSIQ (n = 372)	63.44	6.84
Digit span forward (n = 347)	6.49	1.54
Digit span backward (n = 347)	2.73	1.43
Difficult behavior (SDQ) (n = 374)	12.05	4.17
Prosocial /positive (SDQ) (n = 374)	7.09	1.75
**School achievement scores of the children**		
Reading score (n = 372)	92.06	23.21
Spelling score (n = 372)	85.25	21.89
Math computation score (n = 372)	77.29	15.93

Note: SD: standard deviation: %: percentage; FSIQ: full scale intelligence quotient; SDQ: strength and difficulties questionnaire.

Correlation analysis (S1 Table) showed that outcome variables (reading, spelling and math computation scores) had strong positive correlation with one another viz. reading was correlated with spelling (r = 0.872) and math computation (r = 0.720) and spelling was correlated with math computation (r = 0.735). There were also weak and moderate positive correlations of outcome variables with most of the covariates (except age). The difficult behavior of the children was negatively correlated with the outcomes. There were weak correlations amongst covariates e.g. mothers’ years of education was correlated with FSIQ (r = 0.243) and children’s years of schooling (r = 0.152) and fathers’ education (r = 0.568).

Unadjusted analysis showed that almost all background characteristics had significant association with reading, spelling and math computation performances. Children’s years of schooling was found to be the strongest predictor. Girls scored better than the boys in reading B 4.98 CI (0.27, 9.70) and spelling B 6.32 CI (1.90, 10.75) subscales except in math score B 2.33 CI (-0.91, 5.58). Children studying in private schools scored significantly better in all measurements compared to government schools, whereas children from Madrasa showed poorer performance than the government school children. Parents’ education was also associated with children’s school achievement positively (Table 2).

**Table 2:** Unadjusted and adjusted multiple linear regression analysis of the background characteristics on school achievement scores.

Variables	Reading B (95% CI)		Spelling B (95% CI)		Math B (95% CI)	
	Unadjusted	Adjusted	Unadjusted	Adjusted	Unadjusted	Adjusted
Age	0.16 (-0.75, 0.51)	-0.14 (-0.42, 0.14)	-0.04(-0.36, 0.28)	-0.31 (-0.57, -0.05)*	0.02 (-0.21, 0.25)	-0.19 (-0.38, 0.01)
Sex						
Boys	Reference		Reference		Reference	
Girls	4.98 (0.27, 9.70)*	0.34 (-3.53, 4.21)	6.32 (1.90, 10.75)*	1.92 (-1.69, 5.53)	2.33 (-0.91, 5.58)	-0.47 (-3.22, 2.29)
Years of schooling	11.57 (9.65, 13.50)*	11.28 (9.34, 13.23)*	10.69 (8,87, 12.51)*	10.05 (8.23, 11.86)*	7.44 (6.11, 8.76)*	6.97 (5.58, 8.35)*
Type of school						
Govt. schools	Reference		Reference		Reference	
Private schools	12.09 (5.86, 18.31)*	13.35(8.19, 18.51)*	10.27 (4.45, 16.09)*	10.54 (5.74, 15.35)*	3.82 (-0.46,8.09)	4.03 (0.36, 7.70)*
Madrasa	-5.45 (-13.28, 2.38)	4.81 (-1.50, 11.11)	-9.07 (-16.38, -1.75)*	0.70 (-5.17, 6.58)	-4.21 (-9.58, 1.17)	1.82 (-2.67, 6.32)
Mother’s education (years)	2.60 (1.95,3.26)*	0.89(0.19, 1.59)*	2.65(2.04, 3.26)*	1.18 (0.53, 1.83)*	1.66 (1.2, 2.11)*	0.70 (0.20, 1.20)*
Father’s education (years)	2.62 (2.06, 3.18)*	0.95 (0.34, 1.57)*	2.41 (1.88, 2.95)*	0.62 (0.04, 1.19)*	1.57 (1.17, 1.97)*	0.47 (0.03, 0.91)*
Father’s occupation						
Irregular/died/n	Reference		Reference		Reference	
Regular	11.19 (6.52, 15.87)*	0.24(-4.31, 4.78)	11.37 (6.99, 15.76)*	-0.14 (-4.37, 4.09)	7.30 (4.17–10.60)*	-0.35 (-3.58, 2.89)
Monthly Income						
income ≤ 8500BDT	Reference		Reference		Reference	
Income >8500BDT	10.91 (6.21, 15.57)*	1.47 (-2.92, 5.87)	12.85 (8.52, 17.18)*	4.48 (0.38, 8.58)*	8.17(4.99, 11.35)*	2.75 (-0.38, 5.89)
Housing Index	4.00 (2.21, 5.79)*	0.92 (-0.71, 2.55)	3.72 (2.04, 3.26)*	0.81 (-0.71, 2.32)	3.12 (1.90, 4.34)*	1.07 (-0.08, 2.22)
Crowding index	-2.42 (-3.96, -0.88)*	-0.92 (-2.21, 0 .36)	-2.19 (-3.65, -0.74)*	-0.72 (-1.92, 0.48)	-1.90 (-2.95, -0.84)*	-0.87 (-1.79, 0.04)
Unadjusted R-square	0.454		0.457		0.400	
Adjusted R-square	0.438		0.441		0.383	

CI: Confidence Interval

Note: In the adjusted multiple linear regression model children’s age, sex, years of schooling, type of schools, mother’s education, father’s education, family monthly income, father’s occupation, housing index and crowding index were considered.

*P<0.05

After adjustment of related background characteristics in the model, sex of the children remained no longer significant. Years of schooling still remained in the model as the strongest explanatory factor. Children of private schools showed better scores compared to the children of government schools, however the result for Madrasa student is inconsistent with unadjusted result and they were not statistically significant. Parents’ education remained in the model as an important predictor of children’s school achievement (Table 2).

Unadjusted analysis showed that children’s HAZ and WAZ and mothers’ MUAC and BMI were significantly and positively associated with all subscales of children’s school achievement. But OFC was only associated with reading score. In adjusted model, only WAZ showed positive association with spelling and math computation (Table 3).

**Table 3:** Unadjusted and adjusted multiple linear regression analysis of the anthropometric indices on the school achievement score.

Variables	Reading B (95% CI)		Spelling B (95% CI)		Math B (95% CI)	
	Unadjusted	Adjusted	Unadjusted	Adjusted	Unadjusted	Adjusted
Weight for Age Z score	5.47 (3.30–7.63)*	2.93 (-.09, 5.96)	5.72 (3.69–7.76)*	3.58 (0.74, 6.43)*	4.07 (2.57–5.56)*	3.12 (1.06, 5.18)*
Height for Age Z score	5.67 (3.22–8.12)*	1.99 (-1.30, 5.29)	5.94 (3.64–8.23)*	2.28 (-0.82, 5.38)	3.74 (2.06–5.43)*	0.90 (-1.34, 3.14)
Mother’s MUAC	1.68 (0.97–2.39)*	1.30 (0.11, 2.50)	1.31 (0.64–1.99)*	0.77 (-0.35, 1.90)	0.99 (0.51–1.47)*	0.50 (-0.31, 1.31)
Mother’s BMI	1.17 (0.55–1.79)*	-0.10 (-1.12, 0.92)	1.01 (0.43–1.59)*	0.09 (-0.87, 1.05)	0.78 (0.36–1.2)*	0.11 (-0.59, 0.80)
OFC at cRCT enrollment	1.45 (0.02, 2.88)*	0.41 (-1.07, 1.89)	1.19 (-0.16, 2.53)	0.12 (-1.27, 1.51)	0.87 (-0.11, 1.85)	-0.09 (-1.09, 0.92)
Unadjusted R-square	0.098		0.105		0.101	
Adjusted R-square	0.084		0.092		0.087	

BMI: Body Mass Index, MUAC: Mid Upper Arm Circumference, CI: Confidence Interval, OFC: Occipitofrontal circumference.

Note: In the adjusted model Children’s Weight for Age Z score and Height for Age Z score, Mother’s MUAC and BMI and Children’s OFC at cRCT enrollment were considered.

*p<0.05.

Children’s neurocognitive abilities were found to be significantly associated with better school achievement. However, their difficult behavior was associated with poorer school achievement scores. Among these, children’s digit span backwards was the strongest. These significant associations persisted in the adjusted model. Higher stimulating home environment was associated with all achievement scores, but after adjustment, it only predicted maths computation (Table 4).

**Table 4:** Unadjusted and adjusted multiple linear regression analysis of developmental status on school achievement score.

Variables	Reading B(95% CI)		Spelling B(95% CI)		Math B(95% CI)	
	Unadjusted	Adjusted	Unadjusted	Adjusted	Unadjusted	Adjusted
FSIQ score	1.48 (1.17, 1.79)*	0.60 (0.27, 0.93)*	1.49 (1.20, 1.78)*	0.63 (0.32, 0.94)*	0.90 (0.68, 1.12)*	0.32 (0.07–0.56)*
Digit span forward	6.94 (5.53, 8.35)*	4.28 (2.90, 5.65)*	5.99 (4.63, 7.36)*	2.99 (1.70, 4.28)*	4.40 (3.39, 5.41)*	2.68 (1.66–3.70)*
Digit span backward	8.25 (6.78, 9.72)*	5.07 (3.56, 6.58)*	8.43 (7.08, 9.78)*	5.63 (4.21, 7.04)*	5.25 (4.19, 6.3)*	3.16 (2.04–4.28)*
Difficult Behaviour	-1.15 (-1.71, -0.58)*	-0.67 (-1.14, -0.20)*	-1.04 (-1.58, -0.51)*	-0.53 (-0.97, -0.10)*	-0.52 (-0.92, -0.11)*	-0.17 (-0.51–0.18)
Prosocial/positive behaviour	2.13 (0.77, 3.5)*	1.55 (0.44, 2.66)*	1.78 (0.49, 3.08)*	1.07 (0.04, 2.11)*	1.1 (0.14, 2.07)*	0.71 (-0.11–1.53)
MC-HOME Score	0.70 (0.33, 1.07)*	0.02 (-0.31, 0.34)	0.90 (0.56, 1.25)*	0.19 (-0.11, 0.50)	0.64 (0.39, 0.89)*	0.32 (0.08, 0.56)*
Unadjusted R-square	0.409		0.422		0.334	
Adjusted R-square	0.398		0.412		0.322	

FSIQ: Full Scale Intelligent Quotient; MC-HOME: the Middle Childhood version of Home Observation for Measurement of Environment; CI: confidence interval

Note: In the adjusted model of multiple linear regression analysis children’s FSIQ, score of digit span forward and digit span backward, difficult beahiour, prosocial behaviour and MC-HOME score were considered.

*p<0.05.

Children’s years of schooling and digit span backwards were significant positive predictors of reading, spelling and math computation. This study documented that the young children had higher school achievement scores. Digit span forward positively predicted reading and math while IQ significantly predicted reading and spelling scores. Fathers’ and mothers’ education only predicted reading and spelling, respectively. Reading and spelling performance of the children of private schools was higher than that of the government schools whereas Madrasa children showed insignificant and inconsistent pattern. Children’s prosocial or difficult behaviour and anthropometry were not significantly associated with school achievement except a negative association of difficult behavior with reading score (Table 5).

**Table 5:** Adjusted multiple linear regression analysis of background characteristics, anthropometry indices and developmental status of the children on the school achievement.

Variables	Reading B (95% CI)	Spelling B (95% CI)	Math B (95% CI)
Age	-0.48 (-0.94, -0.02)*	-0.41(-0.88, -0.02)*	-0.47 (-0.80, -0.14)*
Years of schooling	8.09 (5.84, 10.31)*	6.43 (4.33, 8.53)*	5.23 (3.60,6.87)*
Type of school			
Government schools	Reference		
Private schools	10.28 (5.05, 15.51)*	6.22 (1.31, 11.13)*	1.91 (-1.92, 5.73)
Madrasah	1.74 (-4.76, 8.24)	-3.12 (-9.22, 2.99)	0.30 (-0.15, 0.76)
Mother’s education (years)	0.50(-0.20, 1.20)	0.82 (0.16, 1.48)*	0.42 (-0.09, 0.94)
Father’s education (years)	0.68 (0.06, 1.30)*	0.45 (-0.13, 1.03)	0.30 (-0.15, 0.76)
Cognitive development (FSIQ)	0.48 (0.14, 0.81)*	0.50 (0.18, 0.82)*	0.17 (-0.08, 0.42)
Digit span forward	2.38 (0.94, 3.81)*	1.20 (-0.14, 2.55)	1.11 (0.06, 2.16)*
Digit span backward	3.55 (2.01, 5.09)*	4.01 (2.56, 5.46)*	2.49 (1.37, 3.62)*
Difficult behaviour	-0.51 (-0.96, -0.05)*	-0.32 (-0.74, 0.11)	-0.07 (-0.40, 0.26)
Unadjusted R-square	0.583	0.581	0.506
Adjusted R-square	0.547	0.545	0.464

FSIQ: Full Scale Intelligent Quotient; CI: Confidence interval

Note: In the finally adjusted model of multiple regression analysis childre’s age, years of schooling, type of schools, mother’s education, father’s education, children’s FSIQ, score of digit forward and backward and children’s difficult behaviour and intervention at early age were considered

*p<0.05.

## Discussion

The main findings of this study are that younger children, those who attended school for longer years and had better short-term memory had higher reading, spelling and math computation scores. IQ and fathers’ education were predictors of reading and spelling whereas mothers’ education was the predictor of spelling only. Those who attended private schools had higher reading scores.

The study is not readily comparable to other studies because of a number of variations e.g. measurement tools, study participants (population/school based), disparities of background characteristics. We found few studies conducted in developing countries to compare with the findings of this present study. Although we considered many studies to compare with our study findings both in developed and developing countries (9, 15, 21, 31, 32, 47, 49). All the studies were different from each other in many ways e.g. different contexts, disparities of background characteristics, anomaly of outcomes measurement tools.

### Background characteristics and school related factors

We found that the younger children had higher scores of reading, spelling and math computation. Similar results were found in a study among high school students in the USA [31], while older children had higher school achievement in all academic subjects in Ethiopia [9] and India [32] in children of similar age to our study children. It is not clear why this difference exists. It is possible that the tests for older children were more difficult and hence they scored lower, however a study with a larger sample size could clarify the issue.

Parental education and children’s school achievement were found associated in another study in Bangladesh [33] and in other developing countries like Gujrat, India [34] Kenya [35] and Ethiopia [36]. Similar findings were also documented in high income countries. Data of The Programme for International Student Assessment (PISA) study from England, Greece, Hong Kong, the Netherlands, Turkey, and the USA showed that socioeconomic status e.g. parental education, social, and cultural status (ESCS) index had significant effects on mathematics achievement [37]. Even a study using information on six longitudinal data in Europe documented positive association of school achievement with family education [21]. However, our findings are not in line with the findings of the study conducted in Kwara State, Negeria [38], which was conducted in a selected school contrary to our study that was community based and that might have limited variability of socioeconomic status. In Bangladesh it could be concluded that higher socioeconomic group of students were enrolled in kindergartens and in our study 29% students were from kindergartens. Study suggested for early educational intervention to get maximum benefit of child development and school success in low resource settings [39].

Schooling factors i.e. years of schooling and type of school are the dominant explanatory factors of our study. We found that children of private school scored significantly higher than government school for reading competency. This may be due to differences in curricula of the two types of schools. In our settings, private schools usually introduce additional curricula beside government regular curricula that might have had positive impact on school achievement scores. Moreover, children of private schools might have higher socioeconomic status meaning that they are from richer families, which can afford to pay for a private education. Our study findings were similar to other study results which documented that type of school, curriculum, and characteristics of the school correlated with children’s school achievement [15, 17, 40, 41]. It is assumed that when children enter school, school factors become more prominent [42].

### Anthropometry of the children and mothers

These findings of our study were consistent with a rural Peruvian study [43], which was conducted among 588 students aged 12 years from fourth grade of 20 elementary schools in the rural area. The authors of Peruvian study explained that the patterns for height and weight for children of high-altitude areas like Peru may be different than that of children at sea level. There was also no relationship between academic achievement and BMI of fourth grade students predominantly African American children in the USA [44]. One recent systematic review also failed to conclude any relation with higher BMI (obesity) and low academic performance [45]. Although the review established the relationship of obesity and poor academic performance, these findings support that BMI was not a significant predictor of school achievement, which was similar to our findings.

These findings are contrary to the findings of studies from developing countries. HAZ was a significant explanatory variable for school achievement (math computation) in children aged 8–11 years in Ethiopia [9] and 12 to 15 years in Morocco [46]. A study in Sri Lanka also documented stunting as a significant predictor of school achievement in 16,383 students of the whole country who completed grade four [10].

### Neurocognitive behaviours

We reported that children’s school achievement was strongly correlated with Full Scale IQ, digit forward and digit backward at age around 8 years (Table 5). Children’s school performance at 7–9 years was found to depend on brain connectivity at early age, which ultimately results in cognitive development [47]. In Ethiopia, mathematics score was correlated with children’s cognitive development in grade three students [9]. This study used Kaufman Assessment Battery for children (KABC-II) and Ravens colour progressive matrices (Raven’s CPM), while we used the Wechsler abbreviated scale of intelligence II (WASI-II) to measure children’s cognitive development.

IQ was found to be the strongest predictor of academic achievement in many other studies [11] [12, 13, 42]. The relationship between school achievement and IQ was established in both practice and theory [48].

But general cognitive intelligence and school achievement were not correlated in the Iranian [49] and Indian students [50]. The age of the Iranian students who were studying in Malaysia was 18–27 years and age of Indian students was 12–16 years, which is higher than the age of our study population. Influence of cognitive development on school achievement at older age may be mediated by others factors. In fact, the correlation between intelligence and education might be more complex [51].

Cognitive development depends on brain development at early age [47] and brain development is influenced by proper nutrition [52], genetic and environmental contributions [52, 53]. Interestingly, lack of predictability of mathematics by IQ was surprising in our study. This could be related to the educational system in Bangladesh. At the same time using non-standardized IQ test may have been a reason.

Nevertheless, the findings of this study imply that school achievement is not only influenced by a single aspect of education rather by a combination of different sectors. In addition to ongoing education programmes Government should emphasize investing in early childhood development and care to get optimum result from education sector.

Although the nutritional status of children was not an explanatory factor of our study findings, we cannot overlook its impact on school performance since available literature strongly documented association of school performance with nutritional status. Moreover, the link between nutrition and brain development is well established. So, we cannot ignore investment in nutrition sector.

Most of the studies documented cumulative grade point average as school achievement whereas we had used age adjusted word reading, spelling and math computation and reported directly in a communitybased study in rural Bangladesh.

Our use of children’s background characteristics, nutrition status of both children (at early age and at age 8–10 years) and mothers and developmental status of the children in a single study to measure its association with the school achievement in low resource settings is a strength of this study.

Although the study has a good number of strengths, there are some weaknesses also. The study included participants from a sub-district in rural Bangladesh only, so findings may not be applicable to the whole population especially for urban settings of the country. The government and private schools and madrasa used different type of curricula besides the government approved common education system, so our measurement tools may not reflect all part of school achievement of the children although the tools are reliable and were used to measure scholastic performance in our context previously. The unusually low scores of FSIQ, math computation and digit span backwards were of concern, however, one should bear in mind that the tests were not standardized for Bangladeshi children and were only adapted to Bangladeshi context. In another study, similar scores were found in 8-year old children [54]. Considering Bangladeshi children are not exposed to these kinds of tests, the results can be acceptable. Moreover, we did not intend to compare the development and school achievement of these children with Western population. Our aim was to assess factors that were associated with school achievement in Bangladeshi children. Other limitation to this study is that we were unable to collect cultural and geographical factors that influence school achievement. At the end, due to cross sectional nature of the data, the relation cannot be considered as causal. So, the findings of this study must be considered only preliminary.

Better understanding of how school achievement is predicted would inform government to generate evidence-based strategies in low resource settings.

## Supporting information

S1 Fig Reading, spelling and math computation scores by gender, type of schools, father’s education and mothers’ education. (DOCX)

S1 Table Pearson correlation among the variables. (RTF)
