# Supplementary material for: Factors associated with school achievement of children aged 8–10 years in rural Bangladesh: Findings from a post hoc analysis of a community-based study
Source: PLoS One. 2021 Jul 28;16(7):e0254693. doi: 10.1371/journal.pone.0254693 (PMC8318268; doi:10.1371/journal.pone.0254693)
Supplement: S1 Fig — (DOCX) [file pone.0254693.s001.docx]

S1 Figure: Reading, spelling and math computation scores by gender, type of schools, father’s education and mother’s education

**Scores by gender**

**Scores by type of schools**

**Scores by father’s education**

**Scores by mother’s education**
