# Supplementary material for: Factors associated with school achievement of children aged 8–10 years in rural Bangladesh: Findings from a post hoc analysis of a community-based study
Source: PLoS One. 2021 Jul 28;16(7):e0254693. doi: 10.1371/journal.pone.0254693 (PMC8318268; doi:10.1371/journal.pone.0254693)
Supplement: S1 Table — (RTF) [file pone.0254693.s002.rtf]

S1 Table: Pearson correlation among the variables
Variables#	Reading
score	Spelling
score	Math
score	Age of the child (years)	Years of schooling	Type of school	Mother's education (years)	Father's education (years)	FSIQ	Digit span forward	Digit span backward	Difficult behavior	Prosocial behavior	
Reading score	1.000
													
Spelling score	0.872*
	1.000												
Math score	0.720*
	0.735*	1.000											
Age of the child (years)	0.050
	-0.013	0.008	1.000										
Years of schooling	0.536*
	0.527*	0.510*	0.228*	1.000									
Type of school$	<0.001*
	<0.001*	0.038*	0.109	<0.001*	-								
Mother's education (years)	0.378*
	0.408*	0.350*	-0.074	0.152*	<0.001*	1.000							
Father's education (years)	0.430*
	0.421*	0.375*	-0.010	0.229*	<0.001*	0.568*	1.000						
FSIQ##	0.435*
	0.466*	0.385*	-0.041	0.263*	0.040	0.243*	0.267*	1.000					
Digit span forward	0.462*
	0.421*	0.419*	0.066	0.341*	0.170	0.244*	0.226*	0.354*	1.000				
Digit span backward	0.511*
	0.552*	0.465*	0.104	0.368*	0.001*	0.201*	0.230*	0.386*	0.376*	1.000			
Difficult behavior	-0.208*
	-0.200*	-0.134*	-0.038	-0.151*	0.531	-0.200*	-0.165*	-0.128*	-0.021	-0.118*	1.000		
Prosocial behavior	0.163*
	0.144*	0.120*	0.063	0.179*	0.752	0.166*	0.210*	0.064	-0.036	0.074	-0.177*	1.000	
$Association is shown by p-values by one-way ANOVA, *P<0.05
##Full Scale Intelligent Quotient
#We considered the variables for this analysis those were shown in final regression model (table 5)	
